# An immunohistochemical analysis of the neuroprotective effects of memantine, hyperbaric oxygen therapy, and brimonidine after acute ischemia reperfusion injury

**Published:** 2011-04-26

**Authors:** Ulviye Yiğit, Serkan Erdenöz, Ünal Uslu, Ersin Oba, Alev Cumbul, Halil Çağatay, Şamil Aktaş, Emiray Eskicoğlu

**Affiliations:** 1 Bakırköy Dr. Sadi Konuk Training and Research Hospital Clinics of Eye Diseases, Istanbul, Turkey; 2 Akyazı State Hospital, Clinics of Eye Diseases, Sakarya, Turkey; 3 Yeditepe University Medical Faculty, Histology & Embryology Dept, Istanbul, Turkey; 4 Şişli Etfal Training and Research Hospital, Clinics of Eye Diseases, Istanbul, Turkey; 5 Mut State Hospital, Clinics of Eye Diseases, Mersin, Turkey; 6 Istanbul Faculty of Medicine, Istanbul University, Undersea and Hyperbaric Medicine, Istanbul, Turkey; 7 Trakya University, Istanbul Faculty of Medicine, Edirne, Turkey

## Abstract

**Purpose:**

This study applies treatment methods to rat retinas subjected to acute ischemia reperfusion injury and compares the efficacy of memantine, hyperbaric oxygen (HBO) therapy, and brimonidine by histopathological examination.

**Methods:**

Thirty adult Wistar albino rats were divided into five groups after retinal ischemia was induced by elevating the intraocular pressure to 120 mmHg. The groups were as follows: group 1: control; group 2: acute retinal ischemia (ARI) model but without treatment group; group 3: memantine (MEM) treatment group; group 4: HBO therapy group; and group 5: brimonidine treatment (BRI) group. In the control group, right eyes were cannulated with a 30-gauge needle and removed without causing any intraocular pressure change. The ARI group was an acute retinal ischemia model, but without treatment. In the MEM group, animals were given a unique dose of intravenous 25 mg/kg memantine by the tail vein route after inducing ARI. In the HBO group, at 2 h following ARI, HBO treatment was applied for nine days. In the BRI group, a 0.15% brimonidine tartrate eye drop treatment was applied twice a day (BID) for seven days before ARI. Twenty-one days after establishing ischemia reperfusion, the right eyes were enucleated after the cardiac gluteraldehyde perfusion method, and then submitted to histological evaluation.

**Results:**

On average, the total retinal ganglion cell number was 239.93±8.60 in the control group, 125.14±7.18 in the ARI group, 215.89±8.36 in the MEM group, 208.69±2.05 in the HBO group, and 172.27±8.16 in the BRI group. Mean apoptotic indexes in the groups were 1.1±0.35%, 57.71±0.58%, 23.57±1.73%, 15.63±0.58%, and 29.37±2.55%, respectively.

**Conclusions:**

The present study shows that memantine, HBO, and brimonidine therapies were effective in reducing the damage induced by acute ischemia reperfusion in the rat retina. Our study suggests that these treatments had beneficial effects due to neuroprotection, and therefore may be applied in clinical practice.

## Introduction

Central retinal artery occlusion (CRAO) causes severe loss of vision. Treatment trials include massaging of the globe, paracentesis, antiglaucomatous eye drops, and hemodilution or lysis therapy, which in individual cases can improve the visual outcome, although in general the prognosis remains poor.

Acute retinal ischemia (ARI) is a vision-threatening condition encountered in several pathologies, including central and branch retinal artery occlusion, anterior ischemic optic neuropathy, venous occlusive disorders, ocular trauma, and acute angle closure glaucoma [1].

Reperfusion after initial ischemia paradoxically maintains the destruction process, perhaps due to increased levels of extracellular neurotransmitters, reactive oxygen species, and waste products damaging previously unharmed cells during reoxidization [1,2].

Central retinal artery occlusion is a typical example of ARI. Classical and complex treatments have not yet yielded the expected success. Hyperbaric oxygen (HBO) therapy was successfully used in the treatment of central retinal artery occlusion [3,4] and branch retinal artery occlusion, including Susac’s syndrome [5,6]. HBO therapy was reported to be useful in the treatment of ocular vascular diseases [7,8]. Vasoconstriction or vascular occlusion of the retinal vessels is probably a direct response to the interaction between free oxygen radicals and nitric oxide, together with the autoregulation that occurs in this treatment. During HBO therapy, oxygen saturation rises up to 23% and the retina is not damaged [9]. However, the acclaimed HBO therapy has the drawback that it must be applied shortly after the occurrence of the ischemia to be effective. A phase called “ischemic penumbra,” characterized by its reversibility, was described as occurring before definitive ischemic damage [10]. The duration of this critical period is impossible to determine in humans. It is generally admitted that an accurate and rapid therapy must be applied within 24 h. Therefore, practical and urgent therapy is needed, and determining which treatment can provide this was the goal of our study [11-14].

During ischemia, there is activation of the extracellular glutamate-bound N-methyl-D-aspartic acid (NMDA) receptors, which provokes apoptosis by means of intracellular calcium accumulation [15,16]. Memantine, known as a glutamatergic NMDA receptor antagonist, is a derivative of amantadine that inhibits excitotoxicity and neuronal cell death. The protective effect of memantine on retinal ganglion cells (RGCs) has been explained by the presence of NMDA receptors in these cells [17]. Memantine has been used in the treatment of many clinical conditions, including influenza, Parkinson disease and spasticity, Alzheimer disease, and vascular dementia [18]. The neuroprotective properties of memantine have been studied by several laboratories in a large number of in vitro and in vivo animal models, such as in the case of brain stroke, glaucoma, and retinal ischemia–related conditions [19].

Alpha-2a adrenergic receptor agonists are thought to be neuroprotective, preventing RGC death independent of pressure reduction. In vivo studies have shown that in addition to lowering intraocular pressure, alpha-2a adrenergic agonists such as brimonidine decrease RGC death subsequent to increases in intraocular pressure, retinal ischemia, or optic nerve crush. Alpha-2 receptor activation has been implicated in enhanced neuronal survival in glaucomatous in vivo models, and hence alpha-2 agonists are some of the most studied neuroprotective agents [20-23].

The aim of our research was to apply the methods mentioned above to rat retinas subjected to acute ischemia reperfusion injury and to compare the efficacy of memantine, HBO therapy, and brimonidine by histopathological examination.

## Methods

### Subjects

Thirty adult Wistar albino rats at the same age of 24 weeks were used. The rats were housed in the Laboratory of Marmara University Experimental Animal Laboratory under a constant light-dark cycle and were fed ad libitum with normal rat chow and given free access to water. All efforts were made to minimize pain and distress. Protocols were approved by the Şişli Etfal Education and Research Hospital Ethical Animal Care Committee (Permission no. 52/2007).

### Acute retinal ischemia model

Rats were anesthetized intraperitoneally with ketamine hydrochloride (100 mg/kg) and chlorpromazine (25 mg/kg). Pupils were dilated with a topical application of tropicamide 1% (Tropamid; Visufarma, Milan, Italy). Local anesthesia was obtained using proparacaine 0.5% (Alcaine; Alcon Labs., Ft. Worth, TX). The anterior chamber of the right eyes were cannulated with a 30-gauge needle attached to a raised saline reservoir. Retinal ischemia was induced by elevating the intraocular pressure to 120 mmHg. Measurement of intraocular pressure was conducted using a TonoPen XL tonometer (Mentor, Inc., Norwell, MA) calibrated according to the manufacturer’s instructions.

A hand-held ophthalmoscope was used to visually inspect the retinal blood vessels and verify ischemia. After 60 min, the saline reservoir was lowered and the intraocular pressure and retinal circulation were allowed to return to normal over a period of 10 min. The cannula was then removed from the cornea and the animals were allowed to recover.

### Hyperbaric oxygen therapy

For HBO therapy, rats were placed in a small cylindrical monoplace research chamber of 0.6 m^3^. The chamber was flushed with 100% oxygen for 10 min to vent the air inside before the compression. The HBO therapy session consisted of 100% oxygen at 2.5 atmospheres absolute (which is 1.5 atmospheric pressure in addition to normal atmospheric pressure) for 80 min, including 10 min for compression and 10 min for decompression. The first HBO therapy session was performed within 2 h of retinal ischemia. Treatments were conducted three times a day for two days, and then twice a day for seven days

### Wistar rats were divided into five groups (n=6/group)

Group 1: control
Group 2: ARI group
Group 3: Memantine group
Group 4: Hyperbaric oxygen (HBO) treatment group
Group 5: Brimonidine group.
  1. Control group: Right eyes were cannulated with a 30-gauge needle and removed without causing any intraocular pressure change.
  2. ARI group: Acute retinal ischemia model but without treatment.
  3. Memantine group: Animals were given unique dose of intravenous 25 mg/kg Memantine (Ebixa®, Lundbeck, Merz & Co., Frankfurt, Main, Germany) by tail vein route in 1 h after inducing acute retinal ischemia.
  4. HBO group: 2 h following the acute retinal ischemia HBO treatment was applied for nine days at the Department of Underwater and Hyperbaric Medicine Istanbul Faculty of Medicine.
  5. Brimonidine group: 0.15% brimonidine tartrate eye drops (Alphagan, Allergan, Inc. Irvine, California) treatment was applied twice a day for seven days before acute retinal ischemia.

Twenty-one days after establishing ischemia- reperfusion, the right eyes of all rats were enucleated after cardiac gluteraldehid perfusion method, and then submitted to histological evaluation [24,25].

### Sacrifice and cardiac perfusion tissue processing

Rats were deeply anesthetized with ketamine hydrochloride (100 mg/kg) and chlorpromazine (25 mg/kg) and intracardially perfused with 2.5% gluteraldehyde in cacodylate buffer solution (0.1 M cacodylic acid sodium salt, pH 7.4). The eyes were enucleated and immersed in 10% neutral formaldehyde in 0.1 M phosphate buffer (pH 7.4) for postfixation. Paraffin-embedded sections of 20 μm thickness were prepared from the embedded eye tissue.

### Stereological analysis

The optical fractionator method was used to estimate the total number of RGCs. All cells were counted by the same person. In the optical fractionator technique, the optical dissector is combined with the fractionator sampling scheme [26,27].

### Equipment

For quantification, the Stereo Investigator version 7.5 (MicroBrightField, Colchester, VT) was used on a PC system connected to a Leica DM 4000 microscope (Leica, Weltzlar,Germany). A motorized automatic stage was used to control movement in the x,y plane via a connected joystick. Movement in the z-axis was generated manually with the focus button on the microscope and the distance was measured using a Heidenhain electronic microcator (Heidenhain, Traunreut, Germany).

### Delineation of the region of Interest and cell counting

After the section was placed in the microscope, the circumference of the specimen was delineated using a 4× objective lens. The counting was performed using a 63× Plan Apo objective (NA=1.40).

### Sampling

An unbiased estimation of the total number of ganglion cells was obtained from the eye globe by choosing every 10th section according to the systematic random sampling procedure. A sampling area of 900/40,000 µm^2^ was found to be optimal for this study. Dissector height was 16 μm, and a 2 μm zone at the uppermost part of the section was excluded from the analysis at every step as the upper guard zone. Thus, a thickness sampling fraction of 16 μm/t was used, where it represents the mean section thickness.

### Estimate of total cell number

The total number of cells (N) in one eye retina was estimated as outlined by West et al. [26,27] using the equation **n=ΣQ- × 1/tsf ×1/asf ×1/ssf [1], where ΣQ- represents the total number of RGCs counted in all optically sampled fields of the retina; ssf is section sampling fraction (1/10); asf is the area sampling fraction (900/40,000); and tsf is the thickness sampling fraction (defined by dissector height [16 μm]) divided by the estimated mean section thickness.

### Immunohistochemical processing

Serial sections of 20 μm thicknesses of paraffin-embedded blocks were inspected, and medium sections were selected for immunohistochemical procedure. Thirty eye sections stained with an in situ cell death detection kit, POD (Roche Diagnostics; Mannheim, Germany) were used for semiquantitative analyses.

The staining procedure was performed according to the manufacturer’s instructions. Eye sections were deparaffinized and dehydrated. After rinsing in phosphate buffer saline (PBS; 0.1 mM, pH 7.2), the sections were pretreated. For detergent, Triton X-100 (Acros Organics; Geel, Belgium) was employed at 0.1% (v/v) in 0.1% (w/v) sodium citrate (Merck; Darmstadt, Germany) for 2 min on ice, followed by washing of the slides twice in PBS at room temperature (RT). We employed an A Bosch HMT 812C domestic oven operating at a frequency of 2.45 GHz with five power level settings (0–900 W). The best results were obtained with a setting of 4 (600 W). After placing the slides in a plastic jar containing 200 ml of 0.01 M citrate buffer (pH 6), the samples were irradiated them for 45 s, which resulted in an increase in the temperature from RT to 86 °C. At the end of irradiation, an extra 80 ml of distilled water at RT was added to the jar to cool the solution, and the slides were then quickly immersed in PBS at RT (rapid cooling). Background was diminished by preincubating samples with 2% BSA, 10% normal goat serum (Sigma-Aldrich, Taufkirchen, Germany), and 0.03% Triton X-100 in double-distillated water for 30 min at RT. Sections were then treated with the in situ cell death detection kit, POD, and incubated for 60 min at 37 °C in a humidified atmosphere in the dark. Subsequently, the slides were rinsed in PBS three times for 5 min each time. They were then treated for 1 h at RT with a peroxidase-labeled antidigoxigenin sheep Fab fragment (Roche Diagnostics; Mannheim, Germany), followed by washing. Then, 0.05% 3–3′-diaminobenzidine tetrahydrochloride chromogen concentrate and diaminobenzidine substrate buffer (SkyTek, UT) mixture were used for color reaction. Slides were counterstained with Mayer’s hematoxylin.

### Counting

In each sampling frame, the apoptotic, normal, and necrotic cells were marked using three different markers.

### Statistical analysis

All data are expressed as means±standard error of mean (SEM). Parametric test assumptions were available for total RGC number and apoptotic index of the RGC layer (RGCL) of the eye. They were analyzed by one-way ANOVA, and then multiple comparisons between pairs of groups were performed according to Tukey’s test. SPSS version 16.0 system for personal computer (SPSS, Chicago, IL) was used, and p-value <0.05 was considered to be statistically significant.

## Results

**Figure 1:**
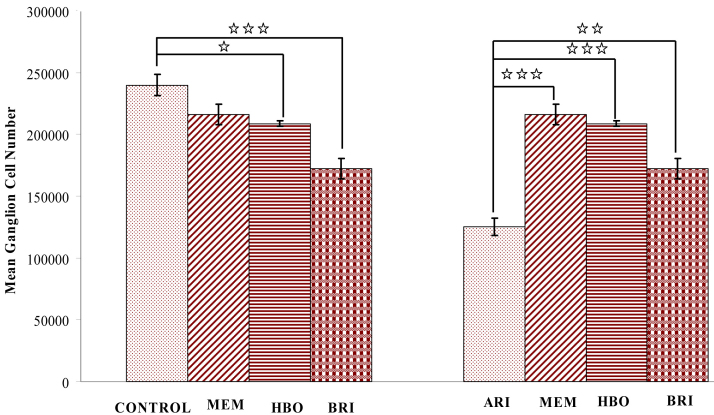
Morphometric analysis of the rat retina shows surviving cells in ganglion cell layer. Left side panel of graph compares to control and treatment groups. There were significant decreases for the surviving cell number of ganglion cell layer in hyperbaric oxygen (HBO) therapy, and brimonidine (BRI) treatment groups. *** p<0.001, *p<0.05. The data showed no significant differences (p<0.05) between the memantine (MEM) treated and control groups. Right side panel of graph compares to the acute retinal ischemia (ARI) and treatment groups. There were significant decreases for the surviving cell number of ganglion cell layer in all treated groups. **p<0.01 and ***p<0.001.

The histomorphometry of the ischemic retina was observed for retinal damage 21 days after ischemia. Severe damage due to high intraocular pressure–induced ischemia was observed in the RGCL, with approximately 52% live cells (125.137±7.184 cells remained, n=6). Cell numbers in the normal retina were 239.926±8.599 in the ganglion cell layer (GCL; n=6, p<0.001). Cell numbers in the MEM group were 215.89±8.36; in the HBO group 208.69±2.05; and in the BRI group 172.27±8.16. Morphometric analysis showed that the percentage of surviving RGCs was 86.9%, 89.9%, and 71.7% compared to postischemic retinas treated with HBO, memantine, and brimonidine (p<0.001 in all treatment methods). Considering RGC counts, there was no statistically significant difference between the control group and the MEM group (p>0.05), but there was a statistically significant difference between the control and the HBO (p<0.05) and BRI groups (p<0.001). There was also a statistically significant difference between the ARI group and MEM group (p<0.001), HBO group (p<0.001), and BRI group (p<0.01; Figure 1 and Table 1).

**Table 1:** General optıcal dıssector.

**Group name**	**Control (n=6)**	**ARI group (n=6)**	**Memantine group (n=6)**	**HBO therapy group (n=6)**	**Brimodine group (n=6)**
Mean total retinal ggl cell count ±SEM	239,926±8,599	125,137±7,184	215,894±8,361	208,687±2,053	172,265±8,158
Dissector particle number	443	231	358	397	326
Section thickness (µm)	19.5	19.5	21.7	19.0	19.3
Number of sampled sections	26.3	23.5	24.3	31.3	26.8
CE	0.05	0.07	0.05	0.05	0.06
CV	0.09	0.14	0.09	0.02	0.12

Mean total ganglion cell count ±SEM, mean dissector number, section thickness, number of sampled sections, coefficient of error, and coefficient of variation of stereological analysis between subjected to ischemia/reperfusion and treatment groups in the rodents.

**Figure 2:**
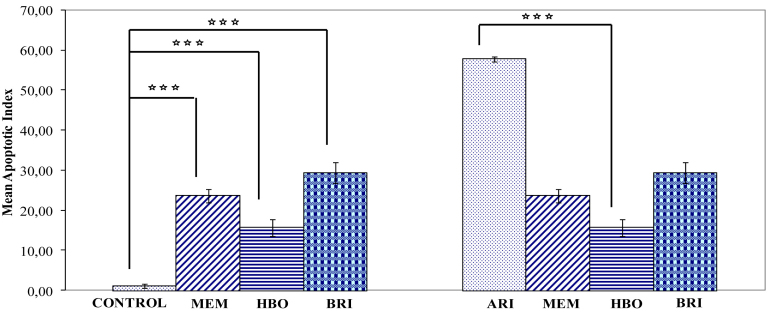
The effect of raised intraocular pressure-induced ischemia for 60 min and reperfusion for 21 days on the percentage of apoptotic (TUNEL positive) cell in rat retina. There is a significant decrease of apoptotic (TUNEL positive) cell (+) cells (***p<0.001) in the control retina compared to treatment groups (MEM, HBO, and BRI) in the left-side graphs. However, there is a significant increase of apoptotic cells (***p<0.001) in retinas subjected to ischemia/reperfusion compared to treatment groups in the right-side graphs. Error bars are ±SEM, where n=6.

**Figure 3:**
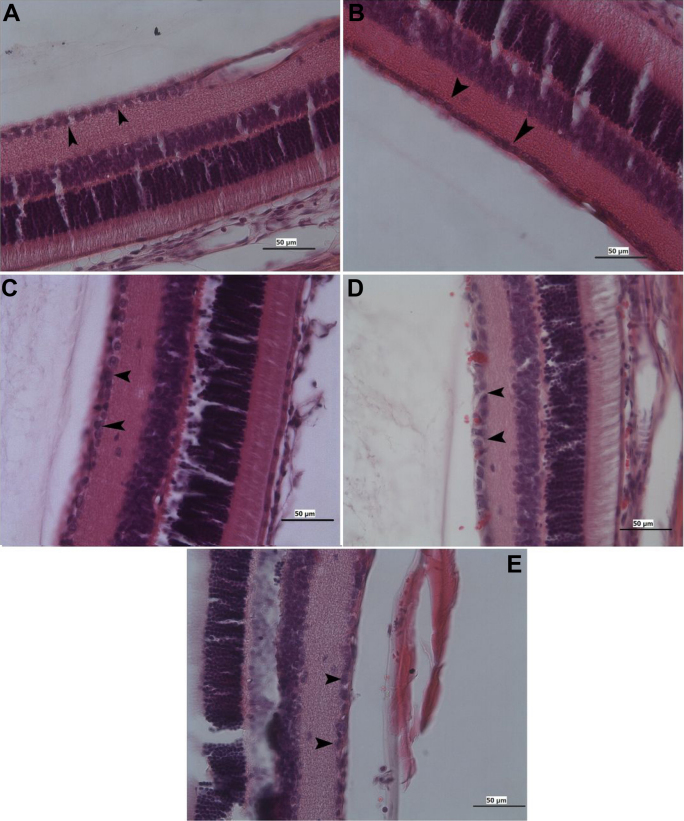
Photomicrographs showing the ganglion cell (black arrow) of the retina from the mature rat. **A**: control, **B**: ARI, **C**: memantine, **D**: HBO therapy, and **E**: brimonidine. Hematoxylin and eosin stain; Bar 50 µm.

**Figure 4:**
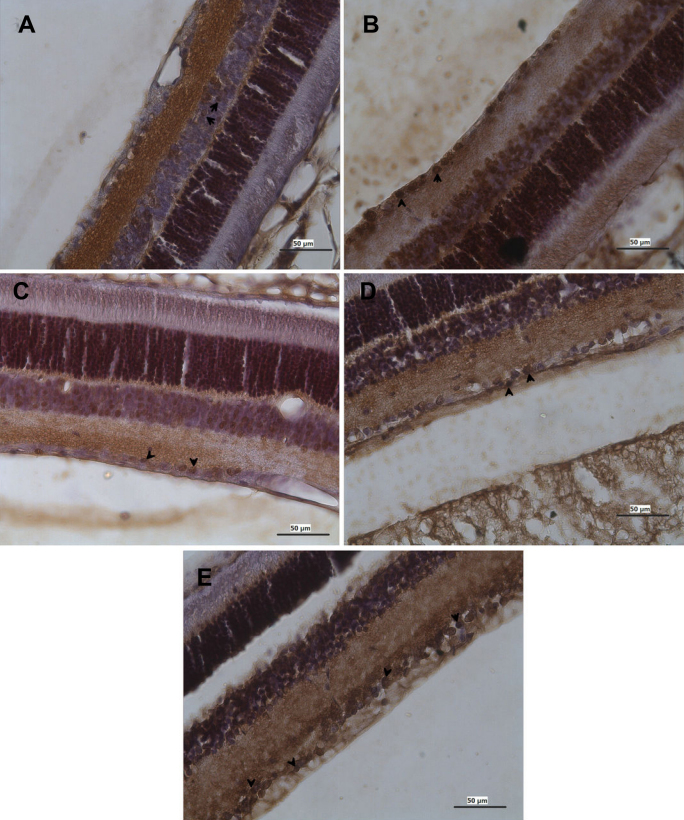
Photomicrographs showing the apoptotic ganglion cell (black arrow) of the retina from the mature rat. **A**; control **B**: ARI, **C**: memantine, **D**: HBO therapy, and **E**: brimonidine. TUNEL+ diaminobenzidine, counter stain hematoxylin; Bar 50 µm.

Terminal Uridine Nick 3′ End Labeling (TUNEL)-positive stained cells were observed only in the GCL. At three weeks postischemia in the ARI group (n=6), the number of TUNEL-positive cells had increased significantly in each section (57.71±0.58% in the ARI group versus 1.1±0.85% in control; p<0.001; n=6). In the HBO therapy group (15.6 ± 0.58%; p<0.001; n=6), MEM treatment group (23.6±1.73%; p<0.001; n=6), and BRI treatment group (29.37±2.55%; p<0.001; n=6), the percentage of TUNEL-positive cells in the RGCL in each section was significantly reduced compared to the ischemic retina. Considering the apoptotic index count, there was a statistically significant difference between the control group and the MEM group (p<0.001), HBO group (p<0.001), and BRI group (p<0.001). There was also a statistically significant difference between the ARI group and the MEM group (p<0.001), but there was no statistically significant difference between the ARI group and HBO group (p>0.05) or BRI group (p>0.05; Figure 2, Figure 3, Figure 4, and Table 2).

**Table 2:** Apoptotic index of the experimental groups.

**Group name**	**Control (n=6)**	**ARI group (n=6)**	**Memantine group (n=6)**	**HBO therapy group (n=6)**	**Brimodine group (n=6)**
Mean apoptotic index percentage (TUNEL [+] cells/total cells) ±SEM	1.1±0.35	57.71±0.58	23.57±1.73	15.63±2.08	29.37±2.55

## Discussion

The present study shows that memantine and HBO therapy were effective in reducing the damage induced by acute ischemia reperfusion in the rat retina. Brimonidine was found to be the least effective therapy. Our study suggests that these treatments had beneficial effects due to neuroprotection, and may therefore be applied in clinical practice.

In this study, the ischemia reperfusion model was obtained through an acute increase of intraocular pressure because in this type of research, this ischemia reperfusion model is widely preferred. Moreover, central retinal artery occlusion may be directly observed by ocular fundus examination in this approach. The validity of our ischemia reperfusion model was proved by comparing the mean RGC count and apoptotic index between the ARI group and control group; a statistically significant difference was found between these groups. This result showed that the ARI model was effective. A bolus intravenous injection of memantine of 25 mg/kg was found to be lethal on rabbits. Smaller doses such as 1–10 mg/kg had neither side effects nor effectiveness [28]. Philip et al. [29] applied 20 mg/kg bolus intravenous injections of memantine on rats and no lethal effect was evident. In our pilot study on rats, we showed that a 25 mg/kg bolus intravenous injection of memantine was not lethal. We can conclude that rabbits are more sensitive than rats. Therefore, we suggest that further clinical studies are needed to investigate doses of intravenous injection of memantine that are effective and not lethal.

CRAO is a typical example of ARI. Since 1859, Van Graefe first described central retinal artery occlusion (CRAO) as an embolic event to the central retinal artery in a patient with endocarditis [10-12], classical and complex treatments have not yet yielded the success expected. The classical proposed HBO therapy has the limitation that it must be applied soon after the occurrence of the ischemia. The generally admitted period in which the therapy will be effective is 6 h, but in humans the duration of this critical period is impossible to determine. It is generally acknowledged that an accurate and rapid therapy must be applied within 24 h. Therefore, a practical, urgent treatment is needed and our study targeted the goal of identifying this treatment [11-14].

Neuroprotection has been a matter of debate for years, since the introduction of experimental ischemia and glaucoma models to the ophthalmology literature. RGCs are a major target for both the quantification of the ischemic damage and evaluation of the therapeutic potential of the agent that is used. It is well established that the death of RGCs secondary to ischemia has been known to occur via the process called apoptosis [30]. An increasing amount of evidence indicates that drugs that show antiapoptotic activity may decrease neuronal death, a major aim of neuroprotective therapy [31,32].

As a result of these considerations, in our study, the percentage of cells undergoing apoptosis was accepted as a main outcome measure, together with the total number of RGCs. To have neuroprotective efficiency, a drug must have properties like good vitreous diffusion, durability, and high concentration value in targeted tissues. Furthermore, it must have correspondent receptor binding sites, presumably on the optic nerve or the retina.

The neuroprotective effects of memantine and topical bunazosin were investigated in an optic nerve ischemia model induced by the delivery of endothelin-1 (ET–1) [33,34]. Memantine, an NMDA antagonist, was shown to be efficacious in terms of the topographic parameters of the optic nerve (cup area, cup depth, and rim volume); in addition, it was found to be neuroprotective in a rabbit model of optic nerve ischemia [33]. Memantine is currently being used in Europe for the treatment of Parkinson disease and spasticity, and it was recently approved in Europe for the treatment of Alzheimer disease and vascular dementia [18].

The neuroprotective effect of memantine has been demonstrated in many studies, especially in terms of acute brain ischemia [34,35]. In ophthalmology, however, contrary to our ARI model, these treatments were essentially applied to chronic glaucoma models in experimental studies. To our knowledge, only a few animal studies about neuroprotective treatments in ARI have been performed [36-38]. The aim of one of the studies was to quantify vitreous amino acid concentrations in pressure-induced retinal ischemia, and to evaluate the neuroprotective effect of memantine administered before and at two time intervals after ischemia [38]. They indicated that memantine reduced ganglion cell loss when given systemically before or within 30 min of retinal ischemia.

Lagrèze et al. [39] induced acute ischemia reperfusion injury in rats and compared the efficacy of cerestat, memantine, and riluzole. As a result, they argued that these drugs have a neuroprotective effect, reducing the excitotoxic damage of retinal neurons. Wolde Mussie et al. [38] investigated the neuroprotective effect of Memantine, an NMDA receptor channel blocker, in two RGC injury models in rats. They found that there was an approximately 80% reduction in RGC number two weeks after the partial optic nerve injury. In that study, memantine (5 mg/kg) caused a twofold increase in compound action potential amplitude and a 1.7 fold increase in the survival of RGCs.

In terms of apoptotic index and RGC count, there was no statistically significant difference in our study between the control group and the MEM group (p>0.05). These results are consistent with those of other studies [23,36-39]. However, a statistically significant difference was found between the ARI group and MEM group. Therefore, we assume that a single bolus dose of memantine may have a neuroprotective effect in ARI.

In view of the apoptotic index, contrary to the statistically significant difference found between the ARI and HBO groups, there was no statistically significant difference between the ARI and MEM groups. We think that these results may be due to cell death originating from a necrotic mechanism more than from apoptosis. In addition, our study showed that memantine has a neuroprotective effect after ARI.

The European Committee of Hyperbaric Medicine determined the indications of HBO therapy. The treatment priority of acute ocular ischemic pathologies is classified as level C, meaning optional. However, in ophthalmological practice, HBO is the first-choice therapy in ARI. In HBO treatment studies on clinical conditions implying ARI, the clinical neuroprotective effect of HBO has been discussed [40,41]. Nevertheless, the efficacy of this method has not been proven immunohistochemically by experimental studies. The major parameters of the studies were visual prognosis and the time lag from the onset of symptoms to the beginning of hyperbaric oxygenation treatment, as well as the time lag until the beginning of retinal reperfusion. Beiran et al. [3] and Weinberger et al. [4] concluded that HBO therapy appears to have a beneficial effect on visual outcome in patients with CRAO.

Our study showed the neuroprotective effects of HBO by immunohistochemically evaluating RGC counts and apoptotic index in an ARI model. The neuroprotective effects of HBO were compared to those of memantine and were found to be similar. In view of the results of our study, we think that HBO therapy seem to be more effective. When comparing the efficacy of memantine and HBO treatments using the same parameters, they show equivalent therapeutic value. We think that the difference in apoptotic index noted between these two groups may come from an unexplained mechanism of apoptosis.

Brimonidine tartrate, an α2-receptor agonist, is an accepted treatment modality for the medical treatment of glaucoma. The α2-receptor agonists have been shown to protect RGCs in experimental models of optic nerve degeneration [42], chronic ocular hypertension [43], transient ischemia [44], and photoreceptor degeneration [45]. Aktas et al. [46] have shown the neuroprotective effect of topically applied BRI in a rabbit model of endothelin-1-induced optic nerve ischemia. As a topical agent, brimonidine was considered to have neuroprotective effects if it could penetrate into the vitreous, maintain its topical application for a given period of time, and have receptors on its target tissues, e.g., the retina and the optic nerve [47].

Based on knowledge about brimonidine receptors and pharmacological concentrations of the drug in the retina, researchers have suggested that the neuroprotective effect of brimonidine may be mediated by vascular modulation or by the upregulation of brain-derived neurotrophic factor in the RGCs [48]. Accordingly, brimonidine treatment was associated with a significant prevention of RGC injury [43]. The effects of brimonidine were reported to include a dose-dependent increase in RGC survival and functioning in a partial crush injury model [20]. In this study, we found a statistically significant difference between the BRI and ARI groups when considering RGC count and apoptotic index. When comparing the HBO and MEM groups with the BRI group, there was no statistically significant difference. In view of these data, further studies need to focus on the modality of the application of brimonidine, along with its duration and doses in ARI treatment.

In conclusion, a single bolus dose of intravenous memantine and HBO therapy were found to be highly effective in the treatment of ARI; however, the topical application of brimonidine was also found to be effective in terms of prophylactic treatment. In this manner, brimonidine seems to be a good choice for preventive therapy for patient at high risk of developing CRAO. Our study shows that when HBO therapy is not immediately available, or some time is needed, as occurs in most cases, memantine and brimonidine seem to be practical and valuable therapeutic and prophylactic agents in acute ischemia. Further and more detailed clinical studies of these two treatments are needed that aim at determining modes of application, doses, and maximum time elapsed after the ischemia period.
